# Genomic Analysis, Progress and Future Perspectives in Dairy Cattle Selection: A Review

**DOI:** 10.3390/ani11030599

**Published:** 2021-02-25

**Authors:** Miguel A. Gutierrez-Reinoso, Pedro M. Aponte, Manuel Garcia-Herreros

**Affiliations:** 1 Facultad de Ciencias Agropecuarias y Recursos Naturales, Carrera de Medicina Veterinaria, Universidad Técnica de Cotopaxi (UTC), Latacunga 05-0150, Ecuador; 2 Laboratorio de Biotecnología Animal, Departamento de Ciencia Animal, Facultad de Ciencias Veterinarias, Universidad de Concepción (UdeC), Chillán 3780000, Chile; 3 Colegio de Ciencias Biológicas y Ambientales (COCIBA), Universidad San Francisco de Quito (USFQ), Quito 170157, Ecuador; 4 Campus Cumbayá, Instituto de Investigaciones en Biomedicina “One-health”, Universidad San Francisco de Quito (USFQ), Quito 170157, Ecuador; 5 Instituto Nacional de Investigação Agrária e Veterinária (INIAV), 2005-048 Santarém, Portugal

**Keywords:** genomic analysis, gene edition, production, reproduction, health, welfare, environment, nutrition, linear types, dairy cattle

## Abstract

**Simple Summary:**

The discovery of the genome has been one of the greatest advances in the development of tools for the genetic improvement of cattle in the last decades. The use of information derived from genomics has been demonstrated to be an efficient alternative for the validation of kinship, adjusted mating, and conservation strategies to generate dairy cattle with desirable traits. However, the impact of the genetic improvement strategies in breeding programs has closed bloodlines in several milk breeds, consequently generating inbreeding depression. Thus, to mitigate the negative effects of inbreeding, several tools associated with genomic analysis are currently available that may be useful to reverse undesirable genetic trends. The present review will provide an understanding of the importance of genomic analysis and how it can be used to mitigate the negative effects of inbreeding depression on different traits related to production, reproduction, health, animal welfare, linear type traits, and adaptability in dairy cattle. A historical overview of genomics and future perspectives will also be covered.

**Abstract:**

Genomics comprises a set of current and valuable technologies implemented as selection tools in dairy cattle commercial breeding programs. The intensive progeny testing for production and reproductive traits based on genomic breeding values (GEBVs) has been crucial to increasing dairy cattle productivity. The knowledge of key genes and haplotypes, including their regulation mechanisms, as markers for productivity traits, may improve the strategies on the present and future for dairy cattle selection. Genome-wide association studies (GWAS) such as quantitative trait loci (QTL), single nucleotide polymorphisms (SNPs), or single-step genomic best linear unbiased prediction (ssGBLUP) methods have already been included in global dairy programs for the estimation of marker-assisted selection-derived effects. The increase in genetic progress based on genomic predicting accuracy has also contributed to the understanding of genetic effects in dairy cattle offspring. However, the crossing within inbred-lines critically increased homozygosis with accumulated negative effects of inbreeding like a decline in reproductive performance. Thus, inaccurate-biased estimations based on empirical-conventional models of dairy production systems face an increased risk of providing suboptimal results derived from errors in the selection of candidates of high genetic merit-based just on low-heritability phenotypic traits. This extends the generation intervals and increases costs due to the significant reduction of genetic gains. The remarkable progress of genomic prediction increases the accurate selection of superior candidates. The scope of the present review is to summarize and discuss the advances and challenges of genomic tools for dairy cattle selection for optimizing breeding programs and controlling negative inbreeding depression effects on productivity and consequently, achieving economic-effective advances in food production efficiency. Particular attention is given to the potential genomic selection-derived results to facilitate precision management on modern dairy farms, including an overview of novel genome editing methodologies as perspectives toward the future.

## 1. Introduction

The discovery of the genome has been one of the greatest advances in the development of tools used for the genetic improvement of cattle in the last decades. The use of high-performance genomics in the selection of dairy cattle represents a remarkable advance to increase the biological and genetic progress of different features and indices in several animal production systems [1].

Recently, several techniques have been developed for the genomic analysis of dairy cows genetic traits related to production, reproduction, health, animal welfare, linear type traits, and adaptability. Several authors highlight the perspectives of using the genomic approach as a crucial genomic selection method for several dairy breeds and stress the great relevance and applicability of this knowledge in breeding programs to incorporate additional alternative features into the traditional pool of traits [2]. In addition to what has been described, several studies reveal an increment in the understanding of the expression and heritability of genotypic traits and indices through the application of genomics for the improvement of production yields and optimization of reproductive programs [3,4,5,6,7]. Currently, the use of information derived from genomic analysis is considered to be an efficient alternative for the validation of kinship, adjusted mating, and conservation strategies for dairy cattle with desirable traits [2]. Consequently, the exchange of reference genomic data among countries has allowed genomic evaluation to increase the accuracy in the prediction of the different traits of interest [8]. However, the impact of genetic improvement strategies in breeding programs has closed bloodlines in several milk breeds, causing a general increase of the inbreeding index which causes an accentuation of the well-known inbreeding depression. The real effect of inbreeding on the deterioration of production and reproductive performance due to the influence of several genotypic features and their expression in phenotypes is still under study. Therefore, to mitigate the negative effects of inbreeding, several tools associated with genomic analysis are currently available that may be useful to reverse undesirable genetic trends.

Although the use of techniques based on genomic analysis has produced important changes in selection and genetic improvement programs in common dairy cattle breeds [9], for these changes to generate genetic improvement, the overall negative effects derived from crossbreeding associated with an increase in the degree of inbreeding must be monitored and managed granting that production yields and reproductive performance are not affected by the inbreeding depression generated. Genomic tools have allowed determining the substantial high-inbreeding level effect over many production and reproductive parameters in high-producing primiparous cows [10]. Therefore, the consideration of the effect of inbreeding depression on the different genotypic and phenotypic characteristics during the planning of selection and genetic improvement programs is crucial. This should be taken with rigor so that inbreeding awareness becomes an integral part of production plans in the dairy cattle industry.

The objective of the present review is to provide an understanding of the importance of genomic analysis and how it can be used to mitigate the negative effects of inbreeding depression on different traits related to production, reproduction, health, animal welfare, linear type traits, and adaptability. From here, we will address several topics covering the use and possible influence of genomic analysis on genotypic traits in dairy cattle as well as its evolution from origins to the present and future perspectives. This document is designed and organized as follows: In the first part, it will briefly describe the chronology and evolution of genomics. In the second part the use of genomics as a predictive tool will be described, as well as the different methodologies used for its application. In the third part, the incidence/impact/effect of genomics as a predictive tool for different parameters associated with production, reproduction, health, animal welfare, evaluation of linear type traits, and adaptability to different production systems and environments will be covered, including causal and highly predictive genetic variants that are key to the prediction of other complex traits.

This review will describe how genomic selection based on genomic analysis represents a promising tool that will improve the predictive accuracy and genetic gain of genomic traits. However, challenges lie ahead in the integration of genomic analysis models covering all the genetic traits and expressing an ideal genomic selection method to adequately regulate inbreeding rates. Therefore, the perspectives to increase genetic gains based on the production and reproductive efficiency, health, animal welfare, and environmental adaptability of dairy cattle will increase the sustainability/sustainability of the production yields in the immediate future as a means for increasing profit in animal production systems.

## 2. Genomics: History and Background

The discovery of genomics and related applications in the dairy cattle industry has led to a significant improvement in the biological prediction of different genotypic traits. Major dairy-producing countries, like United States, Canada, Great Britain, Ireland, New Zealand, Australia, France, the Netherlands, Germany, and the Scandinavian countries have implemented genomic evaluations in their breeding programs, leading to significant changes in the global dairy industry [11].

**Figure 1:**
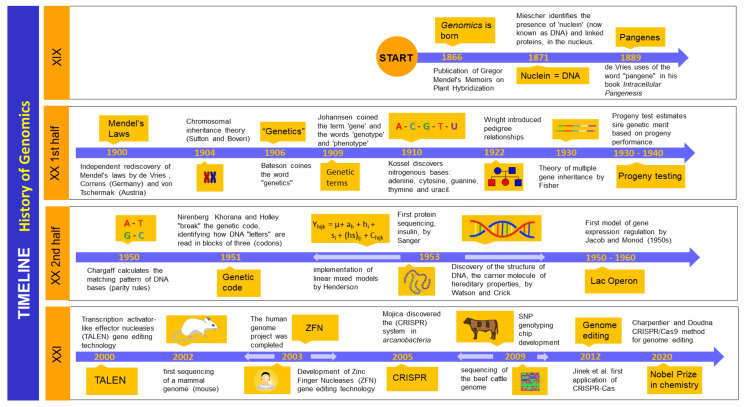
Brief historical evolution of genomics. Origins of classic genetic knowledge are undoubtedly based on Mendel’s discoveries on the inheritance of characters in plants in the XIX century. During the early XX century concepts of genes and chromosome theory became consolidated. Later, during the XX century advances on the chemical basis of inheritance, the breaking of the genetic code and gene regulation discoveries marked the development of genomics and the development of genomic tools in animal science. An impacting development in genomics was the sequencing of the whole human genome in the early XXI century, followed by the ultimate application of this knowledge, the discovery of the gene-editing tools. This led to the awarding of the Nobel Prize to women researchers Charpentier and Doudna in 2020 [14,19,20,21,22,23,24,25,26,27,28,29,30,31,32,33,34,35,36,37].

A brief history of genomics is depicted in Figure 1. In a chronological sequence, genomics became the cutting-edge technology we know today due to its evolution from genome sequencing, including approaches such as genome-wide association (GWAS), whole-genome prediction (WGP), and genome-wide selection of complex traits [12]. At the beginning of the 1990s, the early genetic evaluation identified quantitative trait loci (QTLs) using microsatellite-dispersed markers that correlated with the variation of quantitative traits in the phenotype of a population [13]. Necessary for the development of these technologies for the cattle industry, the first genome sequencing analyses of the bovine species (*Bos taurus*) were carried out in Hereford beef cattle [14]. Later, the genome of animals of the Holstein breed were sequenced as a reference for dairy cattle describing the genome variations compared to the Hereford breed [15]. Around 22,000 genes have been found in bovine species after sequencing analyses, a set of information crucial for future applications in dairy cattle selection [14,15]. In *Bos indicus* the genome was sequenced as well in Gyr, Girolando and Guzerat dairy breeds [16]. The first commercial genotyping chip involving single nucleotide polymorphisms (around 54,000 single nucleotide polymorphisms (SNPs)) was launched in 2007, allowing genomic evaluations in several dairy breeds (Holstein, Jersey, and Brown Swiss) in the US, beginning in 2009, and later in Ayrshire (2013) and Guernsey (2016) breeds [17].

Later, the release of complete genome sequences granted the use of high-performance genetic markers (10,000 to 1,000,000 SNPs), thus improving trait predictions. This interesting technology requires high throughput genotyping platforms (DNA arrays or SNP chips) [12]. In addition, other technologies such as gene expression profile analysis by DNA microarrays (MGEP) began to be widely used in functional genomics or transcriptomics studies. In the near future, genomics/transcriptomics based on SNP chips or microarrays will possibly be replaced by next-generation sequencing (NGS) technologies associated with statistical-computational biology and bioinformatics [18].

There is growing concern about pedigree-based genetic evaluation analyses, as being less efficient and accurate than genomic methods. However, linking pedigree data to information obtained through genomics appears to be advisable, since it may significantly improve the accuracy of predicting traits and indices in dairy breeds [38]. However, there is conflicting work suggesting that genomics per se leads to a faster decline in genetic selection response than phenotypic selection unless new markers and traits are continuously added to the prediction of genetic value [39]. Furthermore, genomic selection of various sires and cows of different dairy breeds, in association with assisted reproductive technologies (ARTs) have significantly increased genetic gain [40]. However, when a single sire is used in breeding selection, genetic diversity significantly decreases despite the above [40]. Furthermore, estimates of variance components from genomic and phenotypic data for one or two generations are less accurate than for three generations, making the calculation of heritability less accurate when using genotypes from selected animals [41]. Moreover, the variance components are unbiased when genomic best linear unbiased prediction (BLUP) also included the data before genomic selection [42]. Thus, it is now always possible to include all genomic and phenotypic data in the analysis to increase the prediction accuracy. Therefore, BLUP and ssGBLUP provide unbiased variances with complete datasets [43].

Today and in the immediate future, genomic evaluation as a functional and everyday tool must also consider the effect of new mutations that can generate a genetic variance for quantitative traits [11]. In this sense, it is essential to integrate additional genes to assess new complex and low heritability traits not yet considered such as those associated with health, food efficiency, metabolism, immunity, and methane emissions, among others [11,44].

Finally, to decipher and associate the functionality of new dairy cattle traits, other ”omic” technologies have been integrated into genomics such as epigenomics, transcriptomics, proteomics, metabolomics, metagenomics, and meta-transcriptomics [5]. Therefore, it is imperative to associate these technologies with physiological, metabolic, behavioral, and environmental factors to improve the accuracy of estimation of current and future traits.

### 2.1. Genomic Factors Related to Production

The incidence of the use of genomic selection in recent years has resulted in a significant increase in production yields for most dairy-related traits in Holstein cattle in countries such as the USA [45]. However, studies involving genetic evaluations of different production traits (milk yield, fat, protein, somatic cells, and longevity) have shown differences between homozygotes/heterozygotes that in the short term could generate variable (high or low) rates of genetic gain [46]. A recent study proposes that genomic selection has been more accurate than pedigree records in the selection of sires and young heifers [47]. Today, sire classification relies on current selection indices such as net lifetime merit (NM$), using a linear combination of approximately 13 traits [48]. Recently, an innovative study established that for milk production traits the genetic effects are constant during lactation [49]. However, the traits may change according to a certain lactation stage, for example, the milk protein content may be affected [50]. Mutations (e.g., F279Y) have also been discovered, for instance within the growth hormone receptor gene showing a strong association with milk, fat, and protein yields [51]. Furthermore, the biological role of at least two causal mutations of genetic loci associated with functional effects on milk, fat, protein, and obesity performance in Holstein cattle has been studied [52].

Analyses of cattle selection pressure to increase milk production yields suggest a positive association with a higher incidence of mastitis [53]. Whole-genome sequences suggest that there are separate genetic variants for the above-mentioned traits. Therefore, genomic selection would allow separating these beneficial and harmful genetic factors through selective breeding in cattle [51]. Accordingly, the genomic parameters associated with production are more heritable than the reproductive ones in Holstein and Jersey cattle, for which building selection indices balancing both types of parameters are recommended [54]. Therefore, and in summary, high density genotyping techniques have improved the association study of the whole dairy cattle genome for several traits. Yet, a series of improving strategies are being proposed to identify more genes responsible for other important economic traits in dairy cattle that will improve yields in the future.

### 2.2. Genomic Factors Related to Reproduction

Fertility rates in dairy cattle play a critical role in breeding programs [55]. Fertility traits are among the most complex, difficult to measure, and modestly heritable [56]. Therefore, determining the benefits of genomic selection in dairy cattle breeds has been a true challenge that will provide relevant knowledge for selection and breeding programs. Recently, advances in genomic techniques have enabled genetic improvement because they increase the accuracy of selection for reproductive traits [7]. Besides, genotyping has helped to resolve existing genetic antagonisms. Interestingly, in Holstein cattle, genomic selection for most fertility and production traits has resulted in a two- and four-fold increase in the rate of genetic improvement, respectively [45]. Thus, genomic selection for fertility indices in sires and young heifers without test records could be used to characterize traits of interest, and consequently, the reliability of the estimated genomic value could be increased if applied to animals with test records [47].

Genomic analyses of most reproductive traits in females show that they tend to be less heritable than in males [7]. However, genomic data indicate that proper haplotype selection in female parents could result in improved male fertility [45]. Conversely, studies applying aggressive selection (high selection pressure) to increase milk production volumes have shown a significant decrease in reproductive performance in the UK and Australian dairy cows [7]. In genomic evaluations, the use of gamete variation parameters in genetic selection programs would lead to significant improvements in genetic progress and the control of genetic diversity [57]. Recently, the use of genomics in several dairy cattle breeds has detected genomic regions of quantitative trait locus (QTL) associated with the gestation period [58], ease of birth traits (paternal and maternal), and perinatal mortality with differences among breeds [59]. This shows that genomic evaluation is an efficient and highly useful tool for the proper selection of animals regarding reproductive traits of zootechnical interest.

### 2.3. Genomic Factors Related to Health and Animal Welfare

In the last decade, genomics has received great attention from the perspective of the study of traits related to animal health and welfare. Hence, several traits such as resistance against infectious and non-infectious diseases as well as adaptability to the environment have been introduced as new selection indices in dairy cattle [60]. Recent studies on the genetic background of immune response traits and immunocompetence have shown relationships with functional and production traits in dairy cattle [60,61]. On the other hand, analyses of genome telomere length have been associated with traits of fitness related to health, survival, and longevity in dairy cows and show that these traits are moderately heritable and highly correlated [62]. Genomic health traits have been used to predict genomic breeding values in Holstein cattle [63].

Several companies managing commercial herds of the American Holstein breed have recently developed and evaluated genomic predictions methods for the estimation of predicted transmission capabilities (heritability) for welfare traits, respiratory disease, diarrhea, and adaptability [64,65]. Given the broad applications of genomics, other studies in Jersey cattle developed genomic predictions for health traits such as mastitis, metritis, placental retention, abomasal displacement, clinical and subclinical ketosis, lameness, and hypocalcemia [63,65,66]. These reports suggest that genomic predictions of calf welfare traits are reliable and comparable to the predictions of other traits of low and high heritability. Thus, genomic predictions for welfare traits represent a key source of information on genetic potential for health of individuals and provide new selection tools to improve dairy production animal welfare [67]. For instance, the genomic trait of well-being in calves and young heifers could be used to effectively predict significant differences applicable to the maintenance of individual animals and herd health [64].

Finally, the epigenetic effect of assisted reproductive technologies (ARTs), such as in vitro embryo production (IVP), has not yet been quantified or evaluated in depth. Much more research is needed to better evaluate and understand genomics and its correlation with IVP potentially affecting bovine health and possible effects during adulthood [68]. Furthermore, identifying and quantifying the traits to include in genomic assessments has proven difficult because the traits are not independent of each other [5]. Overall, all of these studies suggest that the single-trait genomic screening approach may often suffer from antagonistic correlations with traits that are outside the target of selection. That is, choosing multiple traits for genomic evaluations would avoid problems in estimating future productive life in dairy cattle.

### 2.4. Genomic Factors Related to the Environment

The environment, directly and indirectly, plays an important role in animal genomics [69]. Determining how genome–environment interactions work is important to provide relevant information and to better understand the behavior of genes of animal species in different environments. Recently, genomic tests conducted in native versus commercial breeds of cattle showed associations of genes with climatic adaptation, immunity, metabolism, and food safety [70] highlighting genetic correlations between feed efficiency and heritability of production traits in lactating animals [71].

Concerning the above and the current climate change scenario, the bovine species has permanently required substantial modifications in terms of adaptability; however, not all breeds have achieved these levels of adaptation [72]. In the mitochondrial DNA of the ancient genome of *Bos taurus*, genomic introgression was found for the progenitors [73]. This adaptation of the bovine species has largely been human-created, and thus the mixing of the genome (introgression) has been constantly promoted in the process of selecting increasingly productive animals [73]. Consequently, genomic introgression of adapted and non-adapted dairy cattle, whether with low or high production levels, has resulted in a more positive genetic change for both production and adaptation traits [72]. This has not been the case for reproductive traits, which have seen a decrease over the years [74]. Considering this and the specialization of dairy breeds, genomic selection acted on two negatively correlated traits, production (which is moderately heritable) and adaptation (which has low heritability) [72].

One of the major environmental-related advances in genomics over the last decade has been the discovery of traits linked with methane emissions, hypoxia, high altitude adaptation, and heat stress. Several trait association studies primarily emphasize the reduction of negative impacts on the environment [71]. With the use of genomic determination techniques, the prospects for reducing methane emissions will be one of the immediate goals for meeting the challenges of climate change [75].

Comparative analyses regarding the high-altitude adaptation of the genome of domestic yak (*Bos grunniens*) and domestic bovine (*Bos taurus*) show existing genetic differences in protein domains related to sensory perception, energy metabolism, hypoxic stress, and nutritional metabolism [76]. Therefore, most dairy breeds are likely to have little chance to adapt to high altitudes.

The negative effects of heat stress on dairy yields have been noticed [77]. In bovine genome analyses, additive genetic variations related to thermoregulation have been observed in association with candidate genes and functional genes (cellular response to heat stress) for production (milk, fat, and protein) [78]; as well as reproductive traits [79,80]. This would suggest that cows become more sensitive to heat stress as the number of lactations increases and as they age [81]. It should be noted that heat tolerance has not yet been included in selection indices in dairy breeds. Thus, over time, heat tolerance by animals has worsened due to lack of selection pressure [82]. In this way, through genomic analysis, we can select animals with adaptation traits associated with production traits. However, it is essential to continuously test the traits (genomic tests) and seek a balance between them to identify the peculiarities of each dairy cattle breed and thus maintain genetic diversity and evolutionary potential.

### 2.5. Genomic Factors Related to Linear Type Traits

Genomic analysis has allowed the evaluation of several dairy breeds and several reproductive and production traits. However, there are other linear type traits of functional meaning relevant because they are complementary and fundamental for the expression of genomic traits linked to production and reproduction in different production systems [4]. Usually, estimates of individual linear type traits in dairy breed sires have shown moderate genetic relationships regarding the functional life of their offspring. This may vary for specific traits. For instance, heritability is high for udder traits and low for leg and leg set traits [83]. In another study, estimates of genetic parameters made for 23 traits of linear body conformation in the Holstein breed, allowed finding that the heritability estimates were the lowest for the set of rear legs and moderate for body capacity [84]. Another study associated the entire genome with the major loci that affect traits such as height (H), angularity (ANG), and body depth (BD) in the Brown Swiss breed [85]. Furthermore, full genome studies in Holstein cattle showed an association for several traits related to udder health and milking speed, where chromosomes 10–20 and 8–19 were associated with udder index and milking speed and chromosome 13 combined udder index and milking speed [86]. Therefore, a focus on linear conformation traits may be valuable to identify genes that affect economically important traits in dairy cattle.

Genomic analysis of linear conformation indices has been used to characterize several breeds and identify crosses. In the Guernsey breed, performance, functional traits, and linear conformation traits have been evaluated in relation to parental traits [87]. In Holstein cattle the genomic parameters of linear conformation for body condition score (BCS), locomotion (LOC), and angularity (ANG) have shown positive and negative correlations with production traits; however, these traits appear to be genetically independent of other traits [88]. For example, the body condition (BC) has presented significant differences concerning milk coagulation, and consequently, it could be considered as an additive trait to improve yields in cheese production [89]. Furthermore, genomic analysis has shown that the traits associated with locomotion and lameness problems in first lactation cows increases as lactation advances, but the two traits remain relatively constant during the second lactation [90]. Besides, the BC of the animals expressed as low or high BC shows negative genetic correlations with reproductive performance and fertility, and therefore, the BCS could be considered a potential predictor of reproductive status [91].

**Figure 2:**
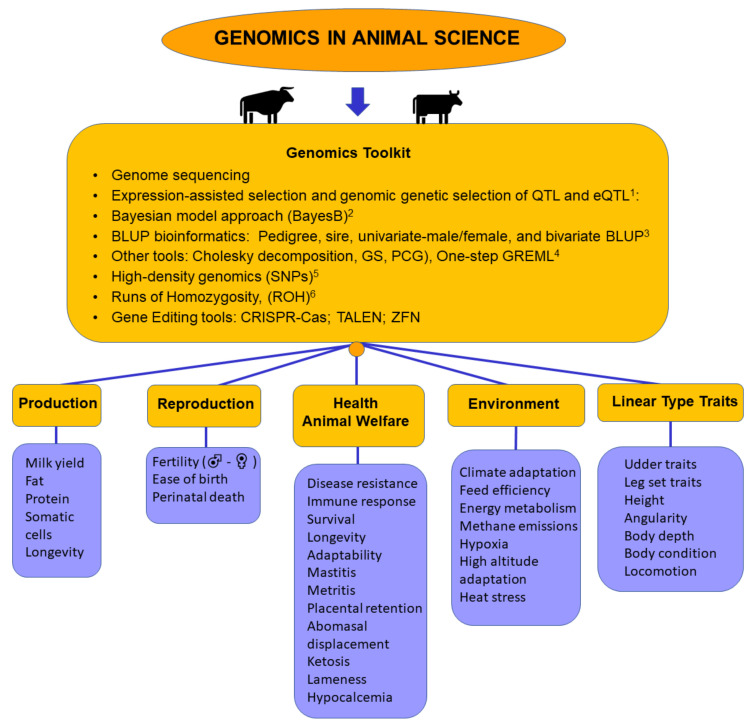
Overview of the impact of genomics on several dairy science topics. (1) Method used for the direct selection of heritable measures of gene expression. (2) A statistical framework for genomic estimations. (3) Genomic relationship matrices for highly accurate estimation of genomic estimated breeding values (GEBV). (4) Unique data resolution methods in very large animal populations; GS = iterative Gauss–Seidel; PCG = preconditioned conjugate gradient; one-step GREML = genome-based restricted maximum likelihood used for >50,000 genotyped animal populations. (5) Analysis of the genetic diversity determining population structure, performing high-density genetic maps and providing genotypes for genome-wide association analysis. (6) Determination of the genomic inbreeding footprint for a specific subpopulation by estimating the individual autozygosity. Under each dairy science topic (production, reproduction, health and animal welfare, environment, linear type traits) examples of relevant traits studied through genomic tools to date are listed.

Genomic selection based on heritable and genetically correlated traits such as linear conformation traits may represent an alternative approach to improve feed conversion efficiency by enhancing the expression of genomic traits in phenotypic traits [92]. Nevertheless, the genetic association and correlation of several specific traits such as methane (CH4) production, body conformation, and health traits have shown low heritability in Holstein dairy cattle [93]. Hence, the use of genomic selection to estimate the functional life of dairy cattle based on conformation traits is possible, but adequate strategies must be proposed to control and regulate the functional traits of productive and reproductive interest for dairy cattle operations. Therefore, genomic selection for linear conformation traits in dairy cattle constitutes an interesting tool that proves the association of multiple traits, increasing the power of the analysis of the complete spectrum of phenotypes that could be affected by variants associated with genomic traits. Thus, the linear conformation indexes could help to simplify the information given for each trait related to new variables. This result could be beneficial for the genetic improvement of the dairy cattle population (Figure 2).

### 2.6. Genomics Toolkit

The use of genomics tools in the context of genetic improvement in cattle plays an important role in animal selection programs [94]. At present, genomic information in dairy breeds is considered fundamental for the operation of breeding programs since it notably contributes to estimating the expected performance of the progeny and the real index of inbreeding in a highly precise and fast way [18].

Different studies over the last decade covering ”systems genetics” or ”systems genomics” approaches have tilted research towards complex traits that have been measured through the implementation of “omics” technologies [95]. Simultaneously, several prediction models have been gradually developed involving normal or Bayesian distributions within each trait by using single-step models to multi-trait, or even more complex analyses such as multi-breeds which have been considered for cross prediction [96]. Notably, one of the greatest advances of the last decades leading to the development of tools for the genetic improvement of cattle was the discovery of the genome [97]. The use of high-performance genomics in cattle selection has shown a remarkable improvement, directing the biological and genetic progress of different traits and indices in various animal production systems [1].

Recently, several genomic analysis techniques have been developed for dairy cattle that are associated with different genetic traits in the fields of production, reproduction, health, animal welfare, linear conformation, and adaptability [98]. Accordingly, genomics has been evolving and providing selection tools for several dairy breeds. Therefore it is perfectly suitable for implementation in genetic improvement programs, incorporating additional and future traits [2]. Other authors pointed out that the application of genomics has increased the understanding of the expression and heritability of genotypic traits and indices to improve production performance, and appropriate practices in reproductive programs [98]. Currently, the use of genomic information has proven to be an efficient methodology in the validation of kinship, adjusted mating, and strategies of conservation of desirable traits in dairy cattle [2].

The accuracy of selection for most high-complexity traits (production, reproductive, and linear conformation traits) can be affected by several factors such as the method of prediction used, the proportion of the categories (sires and cows), or the genetic relationship between traits [99]. Similarly, other authors indicate that discovering the effects of dominance traits regarding genetic additive traits helps to properly understand the genetic variation of complex traits, such as fertility in dairy cattle [100].

Among the potential uses of genomic analysis in dairy cattle breeding, direct selection of heritable measures of gene expression is available and specifically called expression-assisted selection and genomic genetic selection of QTL and eQTL (expression quantitative trait loci) [95]. In this sense, the objective of genomic evaluation on the different genetic traits is to achieve an increasingly accurate selection prediction. For this purpose, different software, sequencing methodologies, and data analysis have been improved, adapted, and implemented. For example, cross-validation methodologies have been applied using the Bayesian model approach (BayesB) and genomic relationship matrices (G-BLUP) [101]. These matrices incorporate new traits and enhance correlation analyses that could replace animal model programs commonly used in traditional genetic evaluations [102]. One of the major advances obtained to date has been the implementation of several computer methods (flexible software), which include models like SNP-BLUP, genomic BLUP (GBLUP), and genomic BLUP of a single step (single-step genomic best linear unbiased prediction, ssGBLUP) to carry out studies of genome-wide association (GWAS), genomic prediction and estimation of parameters, taking into account the index of inbreeding [103,104]. These models implement unique data resolution methods and very large animal populations including finite Cholesky decomposition, iterative Gauss–Seidel (GS), or preconditioned conjugate gradient (PCG) [105]. The one-step GREML technique (genome-based restricted maximum likelihood, GREML) is designed to be applied to populations larger than 50,000 genotyped animals [106].

An improved and more accurate methodology for genomic prediction has recently been proposed for new individuals with a genotype, with and without phenotypes [107]. This methodology recycles parts of the inverse calculations of the coefficient matrix that do not change and combines them with those that do change from one animal to another without re-genotyping the entire population [107]. However, other studies describe that the methodological basis for estimating the index of genetic merit and the accuracy of predictions depends on the number of independent chromosome segments (genomic structure) of the target genome associated with the trait [108]. Definitely, for certain traits, the rate of genetic improvement has almost doubled when SNPs associated with state-of-the-art software are used, and besides, SNPs are being developed for the analysis of different animal populations [109]. In this sense, for instance, the Holstein breed evaluation system could not be useful for the Jersey breed in terms of some traits [110]. Consequently, we propose that genomic systems for the evaluation of dairy cattle cannot be extrapolated for the evaluation of beef cattle and vice versa.

### 2.7. Methods in Genomics

Genomic evaluation is currently present in the leader dairy-producing countries worldwide. The discovery of the genomic analysis in the last years has notably improved production features (genetic merit) in most of the dairy cattle breeds [111]. However, much remains to be done as significant reductions have been observed in the genetic merit of animals for certain traits such as health, reproduction, fitness, and adaptability, which can compromise animal welfare [112]. Consequently, the reliability and accuracy of genomic analysis in breeds such as Holstein can be affected when animals of different origins (sires and dams) or different genetic relationships are included [99].

Although genomic analysis and derived applications have a very important role in the selection of economically relevant traits in dairy cows, the available reports referring to new traits have not yet been clarified in an extensive and detailed manner for specialized meat cattle breeds [113]. In this regard, several studies consider it important to improve predictive methodologies for current genomic traits as well as incorporate new traits that provide high reliability [3,109].

The success of genetic evaluation applying genomic analysis information derived from breeding and selection programs have shown to be an efficient methodology to obtain higher genetic gain rates while reducing selection times and controlling the inbreeding coefficients in dairy cattle populations [98]. Likewise, analyses of genetic diversity in various Irish cattle dairy breeds by using SNPs considered the correlations with the different coefficients of genomic inbreeding and pedigree to maximize heterosis in breeding strategies [114]. The most accurate estimate of the inbreeding rate can be associated with the results obtained from the analysis of genomic data [115]. All research about this methodological framework describes genomics as a fundamental tool for understanding the functionality of genetic traits in dairy cattle. Thus, for instance, commercial individuals of the Holstein cattle breed in the USA have been studied and genetic predictions on health and welfare traits (placental retention, metritis, ketosis, abomasum displacement, mastitis, and lameness) have been satisfactorily obtained through genomics. Furthermore, analyses of the welfare traits in young cattle have been reported that can be used to effectively predict significant differences in future individual health performance [64]. In recent years, genomic analysis has also allowed us to understand the degree of association between various traits within a specific individual and within different populations, and therefore determine associations of genomic regions with other traits to appreciate their expression, evolution, and prospects for future performance [116]. Recently several studies described such associations between production, reproductive and other traits. For example, genomic predictions have been estimated for spermatogenic functional fertility traits [117] associated with age at puberty, age at first birth, and gestation status traits in animals [118].

Recently, many applications of genomics have emerged in the animal science field. Several countries leading in genotyping, including the United States through its Department of Agriculture (USDA), have already begun to conduct genomic evaluations of production and health traits in Holstein, Jersey, and Brown Swiss breeds [119]. Furthermore, genomics of mitochondrial DNA has allowed the classification of animals into specific subspecies and genetic lines [13]. A recent study shows reduced efficiency and low reliability of genomic prediction in small populations of dairy cattle [120]. However, other authors, using high-density genomics (SNPs), express that genomic selection of several breeds has increased the accuracy of genomic evaluation in small population breeds that do not have reference populations [121]. Regardless of the reference population, adding more genotyped animals to genomic evaluations would significantly increase genetic gain and reduce the rate of inbreeding in the offspring [122]. Accordingly, high-density genotypes, reference genotypes, and those already available from several dairy breeds would improve the accuracy of estimation of genomic evaluations of breeds with small populations [123]. Therefore, the genotyped candidate animal without progeny may also contribute information to genomic evaluations, affecting the estimation of other traits as well [124].

In recent years, several studies dealing with the genotyping of continuous homozygous segments (runs of homozygosity, ROH) have been generated, providing a new way to assess the degree of genomic inbreeding [125]. In addition, when very little data is available for genotyping analysis, several strategies are suggested for the genomic evaluation in dairy breeds such as pedigree BLUP, sire-model genomic BLUP, genomic BLUP univariate-male/female, and also genomic BLUP bivariate to increase the accuracy of genomic estimated breeding values (GEBV); [126]. This indicates that genotyped females would improve the accuracy of GEBV concerning the progeny records of the sires evaluated.

Several studies have used genomic selection in high-yielding Holstein and Jersey cows to determine the degree of association of the components of additive variation and dominance for fertility and milk production traits [127,128,129], and to identify dominance effects for production traits but not for fertility traits [100]. Among the major advances in genomic analysis, the development and standardization of the genomic evaluation system based on SNPs have been described. This system has been largely introduced for the analysis of bovine embryo biopsies with a precision similar to that of an adult to minimize the generation intervals as much as possible [109]. Likewise, SNPs have allowed the identification of haplotypes with different selective pressures and evolutionary patterns likely due to a diverse genomic selection process produced during cattle domestication, breed formation, and recent genetic improvement [130]. Finally, several studies have concluded that genetic gain has considerably increased through the application of genomic selection [8]. However, simultaneously to genetic gain, a significant growth in inbreeding rates has been observed in certain breeds such as Holstein and not in others such as Montbéliarde and Normande [40]. All the above references are intended to provide a fundamental understanding of the impact of genomic evaluation on various current and future traits in dairy breeds.

### 2.8. Emerging Genomics: Nutritional, Metabolic, and Environmental Genomics

The genomics of nutritional and metabolic traits related to the environment opens possibilities to develop novel concepts for production functionality in dairy cattle populations. Understanding the mechanisms through which genes are expressed and interact with the environment is of remarkable importance because it allows correcting undesirable features and including other traits of zootechnical interest.

Genomic analysis has recently proven to be an efficient methodology to estimate dairy cattle traits not traditionally considered important. Consequently, the interest in production traits in the dairy industry has led several researchers to study specific related genes. For example, several genes have been described that are differentially expressed during lactation and participate in biological processes such as the development of the mammary gland, protein and lipid metabolism, signal transduction or cell growth, and differentiation [61]. Other studies based on genomic analyses have correlated energy balance traits expressed as energy/protein ratio, somatic cell count, and mastitis to improve the prediction of health traits in primiparous dairy cows. Furthermore, several authors suggest that these traits should always be included in selection and genetic improvement programs [131]. On the other hand, regions of the genome have also been identified as involved in the regulation of the immune system and the several metabolic processes in Guernsey, Jersey, and Holstein cattle [132]. Besides, other genomic regions associated with ketosis susceptibility (metabolic health) have been identified in Jersey cattle [133], as well as regions related to additive genetic variation of the incidence of milk fever [134].

Regarding metabolic activity, genotyping work has been done in Holstein dairy cattle based on the number of births (first, second, and third births) highlighting the existence of high genetic correlations between non-protein nitrogen (MUN) and several production [135] and reproductive traits [136]. In addition, genomic selection for body energy traits and blood metabolites could facilitate genetic improvement of fertility and overall reproductive efficiency of dairy cattle [137]. A new role for urea in reducing egg competition and modifying embryonic gene expression has also been observed [138].

In countries at the forefront of genomic evaluation, such as Australia, new traits such as feed efficiency and heat tolerance have been included in association with health traits [139]. Thus, to understand the biology of feed efficiency, genomic selection has incorporated residual feed intake (RFI) as an important economic and environmental trait that has been linked to production levels, feed costs, and methane emissions [140]. This is reinforced by studies that indicate that genomic information has increased predictive reliability for RFI enhancement compared to the exclusive use of pedigree information [141]. Consequently, the appropriate approach to genomic selection lies in the understanding that it represents an important tool for introducing and associating new performance-oriented traits in dairy cattle breeding.

Finally, some authors have established genomic reliabilities for different traits evaluated in dairy cattle breeds between 60 and 75%, and in new traits, they could be under 50% [139,142,143]. Consequently, the lower genomic reliability of new traits could reduce the overall reliability of the index [139]. In conclusion, improving the reliability and accuracy of predicting traits could include phenotypic data from reference populations, sequence data, and gene expression studies (Table 1).

## 3. Futuristic Genomics: Gene Edition

Genome editing comprises a set of genetic engineering tools that allow the direct manipulation of a sequence in the genome of an organism by removing, inserting, or replacing nucleotides for scientific or commercial purposes. This technology employs nucleases (enzymes coined as “molecular scissors”), which hydrolyze or catalyze the double-stranded DNA in a very precise way at specific sites in the genome [148]. Genome editing has transformed genomics in the last decade, incorporating CRISPR (clustered regularly interspaced short palindromic repeat), a technology application based on the prerequisite knowledge of the genes and the whole genome [149]. CRISPR sequences form the basis of the CRISPR-Cas adaptive immune systems of prokaryotes [150]. This technology was developed during the process of understanding the properties of this unique defense mechanism in arcanobacteria [150]. Thus in 2012, the potential of CRISPR-Cas systems to trigger specific genetic modifications that can be applied to virtually any organism was discovered, and a year later, it was successfully applied to make specific modifications in the genome of mammalian species [150]. This technology has become one of the most promising tools in human medicine and animal, plant, and microorganism biotechnology [151] and allows the efficient engineering of the genome by targeting either individual cells, whole organisms, or both, while controlling transcription or modifying the epigenome [152]. Furthermore, CRISPR technology is starting to be used in genetic improvement programs for dairy cattle [153]. Although genome editing has led to fundamental genetic changes, the greatest attention is being given to the use of CRISPR-Cas9 due to its application in the genetic modification of germ cells (genomic editing of the germline) and human embryos, as well as in studies of the interaction of the genome with the environment, agriculture, and livestock issues [154].

The Cas9 protein is derived from the CRISPR Type II bacterial immune systems (CRISPR/Cas9) that can be easily programmed to target new sites by altering the RNA guide sequence [155]. Recently, several novel types of systems associated with single polypeptide CRISPR have been discovered, including Cas12a/Cpf1 and Cas13a/C2c2 [156]. These additional systems have unique structural and functional characteristics, which provide new opportunities for applications in genome editing, including nuclease regulation and delivery, target specificity, and host repair diversity [148]. Therefore, the development CRISPR-Cas-based tools have made the editing of specific gene sequences easier and more reliable. For example, in Japanese black cattle (Wagyu) there is a recessive type of pathology called isoleucyl-tRNA synthase syndrome (IARS), and thanks to the use of CRISPR-Cas9-assisted genome editing, a single mutated nucleotide in the IARS gene was replaced with the appropriate nucleotide to successfully correct this mutation [157].

Another cutting-edge technology in the field of genome editing besides CRISPR involves the transcription-activating nuclease (TALEN). This system can be artificially engineered to bind to a chosen DNA sequence, allowing it to be cut at desired locations for use in genomic editing or in situ genome editing, a capability shared with other nucleases like zinc finger nucleases (ZFN) that make use of the target proteins that bind to the DNA at specific locations [155].

Homologous recombination has been used to edit genomes in different animal species [158]. One example is the genetic modification of pigs as disease models or as organ donors for xenotransplantation targeted for humans [159]. Examples involving other farm animals include goats that produce human lactoferrin [160] and cattle that produce human serum albumin in milk instead of cow native milk proteins [161]. Antiviral proteins such as lysostaphin [78] and human lysozyme in the mammary gland [162] have also been successfully developed in bovine species. Furthermore, in dairy cattle, hornless animals [163] and non-allergenic dairy animals have been obtained [164]. Among other zootechnical applications, genome editing could help dairy cattle to better adapt to environmental conditions or specific production systems, which could improve fertility, growth, health, and animal welfare in herds [153]. Besides, it would allow the introduction of beneficial alleles such as those for heat tolerance or disease resistance into dairy cattle breeds while maintaining or even accelerating the rate of genetic gain already achieved by conventional breeding programs [165].

Regulatory perspectives may slow the widespread implementation of genome editing in animals. Several international organizations consider that genome editing, used to replicate a “natural” mutation, should not be of regulatory concern because it would be equivalent to natural zootechnical management of existing alleles from the natural gene pool. On the contrary, the US FDA considers that there is a need for regulatory oversight of intentional genomics in animals because the editing of one target allele or nucleotide may indirectly or accidentally result in editing another with potentially detrimental effects. For example, there could be modifications affecting food safety due to changes in protein expression, interruption of protein functions, protein over-expression (e.g., hormone receptors, etc.), or the creation of new expression products [166]. Therefore, it is important to note that the FDA has integrated the regulation of intentional genomic alterations in animals into its veterinary regulatory program [167,168]. However, focusing specifically on dairy cattle, CRISPER technology has the potential to either modify, restore, or both, genetic diversity by allowing dairy cattle genetic resources to be used effectively. Furthermore, genome editing technologies can reduce costs in the long term while meeting animal health requirements in animal breeding and contribute to the creation of new biological industries. From their origins, studies based on gene editing technologies are exponentially expanding, suggesting that the associated applications could avoid the loss of excellent genomic resources in dairy cattle. For example, these technologies could develop into innovative methods for faster progress in the selection, breeding, and improvement of desirable production and reproductive traits in dairy cattle in the future. Moreover, the incorporation of other technologies such as nanotechnology, stem cell therapies, and advanced bioinformatics software could boost the potentials of genome editing tools such as CRISPR and open new horizons in the manipulation of the genome in the animal science context.

## 4. Conclusions

The application of genomic analysis in dairy cattle selection provides very interesting predictions for the genetic improvement in the short/medium term while controlling the inbreeding coefficient, reducing the generational interval, and improving the selection of progenitors, thus generating more productive genetic lines. The estimation of the different traits in dairy cattle is more efficient through the use of data derived from genomic analysis than through data derived from the pedigree. However, the association of both methodologies significantly improves the estimates in terms of prediction and accuracy. Genomic trait estimation accuracy must be supported by multi-generation databases to make predictions more consistent. This will be most noteworthy with the incorporation of new traits into the evaluations, considerably improving selection programs based on such estimates at both the individual and population levels. Selection rates based on genomic analysis provide a better understanding of the biology of the animals, new sources of data, novel genome editing methodologies, and pertinence for changing economic and environmental conditions. This becomes highly relevant as estimations derived from dairy cattle genomic data regarding adaptability traits in different environments or settings are more needed than ever. Genomic methodologies are a perfect fit during these challenging times of global climate change.

Dairy cattle genomic-based selection will be used in the very near future for genetic selection in the beef cattle industry and other animal species. The frequency of rare alleles, recessive genes, and new mutations could generate genetic variability of desirable economic traits. Thus, the information derived from genomic analysis represents an important alternative for the production of databases of great interest in animal sciences and novel genome editing applications, accelerating the process of genetic evaluations in dairy cattle.

## Figures and Tables

**Table 1:** Comparison of methods by pedigree and genomic tools for the estimation of genetic merit in dairy cattle

Trait	Prediction by Pedigree Tools	Prediction by Genomic Tools
Reliability	High, depending on the size of the progeny studied (between 46.00 and 72.00%) [144]	Very high (between 73.30 and 93.50%) [145]
Time to obtain predictive data (Progeny Testing)	High (A long waiting period must be expected to have sufficient production data in children and grandchildren that in animals of long generations like cattle can take years) [96]	Very low. A sample of ear cartilage tissue (DNA) is sufficient and the waiting time for the laboratory results is relatively short. Animals can be genotyped as early as being newborns [96]
Time involved	High, because it requires taking and analyzing production data from generations on a large number of daughters and granddaughters [11]	Low [11]
Cost	Low-medium [11]	High (but constantly decreasing as technology advances) [11]
Modeling with unknown parent groups to model differences in genetic merit with 0.3 and 0.1 heritability accuracy	Estimation of minor and more biased predictions. Suggests poor estimates of genetic trends despite having little bias for validations in young genotyped animals [146]	Estimates accurate and unbiased predictions for young animals and, at the same time, adequately considers genetic trends [146]
Estimation of genetic variance and genetic merit variance predicted through the use of genome ratio or pedigree matrices	Reliability: young bulls; without pedigree: 0.00; known sire: 0.22; with full pedigree: 0.35 [147]	Reliability: young bulls; without pedigree: 0.48; known sire: 0.58; with full pedigree: 0.68 [147]

Pedigree and genomically obtained prediction data can be used in combination. This is highly desirable to obtain higher accuracy in the estimation of genetic merit. As daughter information gradually becomes available, it may be included in the bull’s genetic evaluation, and the reliability of the bull’s data will tend to increase. [11,96,144,145,146,147].
